# Supplementary material for: A New Method of Artificial-Intelligence-Based Automatic Identification of Lymphovascular Invasion in Urothelial Carcinomas
Source: Diagnostics (Basel). 2024 Feb 16;14(4):432. doi: 10.3390/diagnostics14040432 (PMC10888137; doi:10.3390/diagnostics14040432)
Supplement: Supplementary file 1 [file diagnostics-14-00432-s001.zip › diagnostics-2861533-supplementary.pdf]

diagnostics

Routine histopathological processing of the tissue:

- All the fragments were put in cassettes and immersed in 10% buffered formalin until next day (18-24 hours).
- Further routine histopathological processing to paraffin with Leica Peloris 3 automatic tissue processors (see Table S1 supplementary material) was performed.
- The tissue was embedded in paraffin blocks (embedding stations Thermo Fisher Microm EC 1150 H, Leica EG 1150H, Sakura Tissue Tek and Leica Arcadia).
- The paraffin blocks were sectioned at 3 microns thick (semi-automated Rotary Microtome Leica RM2255 and RM2265).
- The resulting slides were stained with H&E on a Leica Spectra staining station.
- After diagnosis, the slides were kept in the Colentina University Hospital archive.

Table S1: Processing protocol for automatic tissue processor Leica Peloris 2

No.	Reagent	Time	Temperature	Pressure/ Vacuum	Time of draining
1.	Formalin 10%	44 min	45°C	P	10 sec
2.	Ethanol100°	30 min	45°C	P	10 sec
3.	Ethanol 100°	60 min	45°C	P	10 sec
4.	Ethanol 100°	60 min	45°C	P	10 sec
5.	Ethanol 100°	60 min	45°C	P	10 sec
6.	Ethanol 100°	90 min	45°C	P	10 sec
7.	Ethanol 100°	90 min	45°C	P	10 sec
8.	Xylene	75 min	45°C	P	10 sec
9.	Xylene	75 min	45°C	P	10 sec
10.	Xylene	210 min	45°C	P	10 sec
11.	Paraffin	120 min	60°C	V	10 sec
12.	Paraffin	180 min	60°C	V	10 sec
13.	Paraffin	180 min	60°C	V	10 sec

TableS2: Experience of pathologists performing annotations

Pathologist	Grade	Years of expertise as certified pathologist
SZ	Senior pathologist, PhD, professor of pathology	24
CP	Senior pathologist, PhD	13
LN	Senior pathologist, PhD, lecturer	15
MiC	Senior pathologist, PhD student	8
LS	Senior pathologist, PhD	10
AC	Pathologist, PhD student	5
MB	Pathologist	5
OS	Pathologist	1
IT	Pathologist	1
AV	Pathologist, PhD student	0

Annotation process

The ROIs were completely annotated ("pixel per pixel annotations"). The pathologists annotated manually for 21 classes: high-grade tumor, low-grade tumor, adenocarcinomatous differentiation, squamous differentiation, tumor retraction, empty spaces within the tumor, necrosis, invasion, LVI, electrocoagulation, stroma, vessels, inflammation, hemorrhage, smooth muscle, non-tumoral urothelium, normal urothelium, reactive urothelium, von Brunn nests, nondiagnostic, no tissue.

"No tissue" class was used to annotate empty space around fragments of tissue (spaces within the tissue — i.e., empty space within the tumor, tumor retraction spaces, empty vascular lumen, etc., were not included in this class).

"Nondiagnostic"' class was used to annotate areas of tissue with considerable damage due to electrocoagulation, which are practically impossible to identify (namely tissue too burned to be diagnosed). Mild electrocoagulation artefacts do not always hamper the diagnosis (i.e. an area of high-grade tumor with mild electrocoagulation can still be diagnosed correctly; a low-grade tumor that is mildly electrocoagulated is prone to a differential diagnosis with high-grade tumor).

These classes were grouped as follows: major classes (classes that are mandatory — every annotation must belong to a major class), minor classes (an annotation from a certain major class may or may not have a minor class) and a special class with exceptional status – LVI

A. Major classes are: high-grade tumor, low-grade tumor, stroma, smooth muscle, nontumoral urothelium, nondiagnostic, no tissue. Any structure in a ROI must belong to one of these categories and they are mutually exclusive.

B. Minor classes are lesions/ structures/ specific patterns that are correlated with tumor, stroma, smooth muscle and nontumor urothelium, with only one of them, few of them, or all of them. For instance:

- electrocoagulation (burning of the tissue during resection) can afflict any part of the tissue.
- inflammation can afflict any part of the tissue except nondiagnostic tissue.
- adenocarcinomatous differentiation, squamous differentiation, necrosis, empty space within the tumor, tumor retraction, and invasion are associated only with a tumor.
- vessels are associated only with stroma.

C. "LVI" are particular structures defined as a tumor within the vessel. This class has exceptional status because usually "tumor" and "stroma" are mutually exclusive classes, except for LVI.

It is virtually impossible to obtain perfect pixel per pixel superposed adjacent annotation areas. One will have either superpositions or gaps. The pathologists were advised to avoid gaps, the superpositions being later corrected automatically. Thus, the annotations were made with superposition of adjacent classes; one of them, for example a high-grade tumor, was drawn with the utmost precision, while the adjacent stroma was superposed on the tumor; further analysis of the date performed automatic subtraction of class "stroma" from the pixels with double designation of "high-grade tumor" and "stroma". We establish the order in which the conflicts will be solved in favor of first class: no tissue > high-grade tumor> low-grade tumor> nontumor urothelium > smooth muscle> stroma> nondiagnostic.

Table S3: Performance metrics used to evaluate our proposed method

Performance metrics	Formula*
Sensitivity	TP / (FN + TP)
Specificity	TN / (FP + TN)
Accuracy	(TP + TN) / (TP + TN + FP + FN)
F1	2TP/(2TP + FP + FN)

TP – true positive, TN – true negative, FP – false positive, FN – false negative

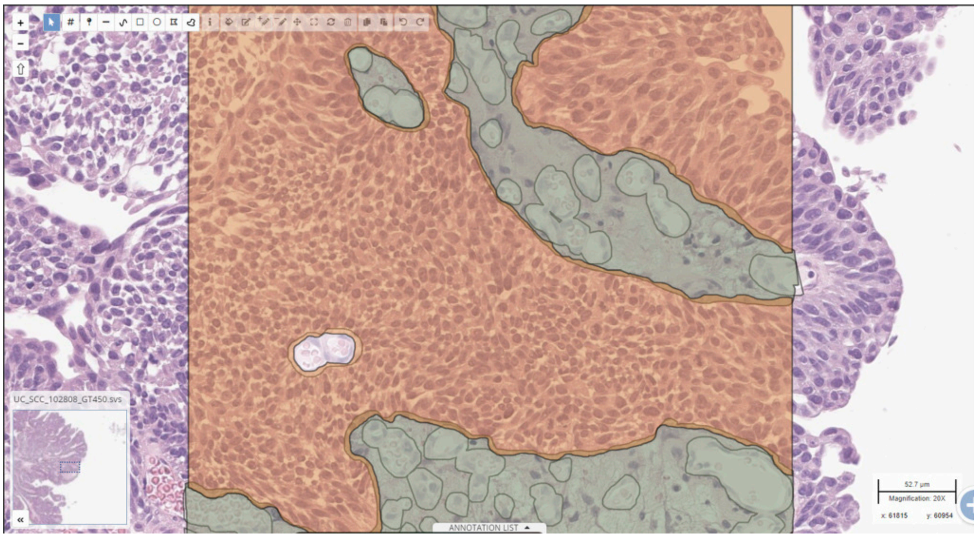

Figure S1. High-grade tumor annotations — high-grade tumor in orange; stroma in green is overlapped on the tumor. HE 20x

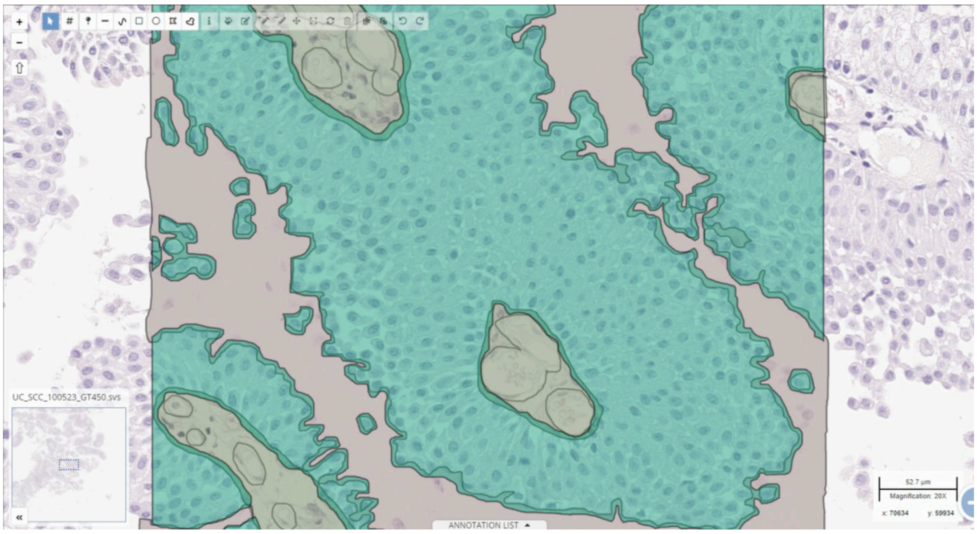

Figure S2. Low-grade tumor annotations — low-grade tumor in light green; stroma in green is overlapped on the tumor; “no tissue” in grey with low-grade tumor overlapped on the no tissue class. HE 20x

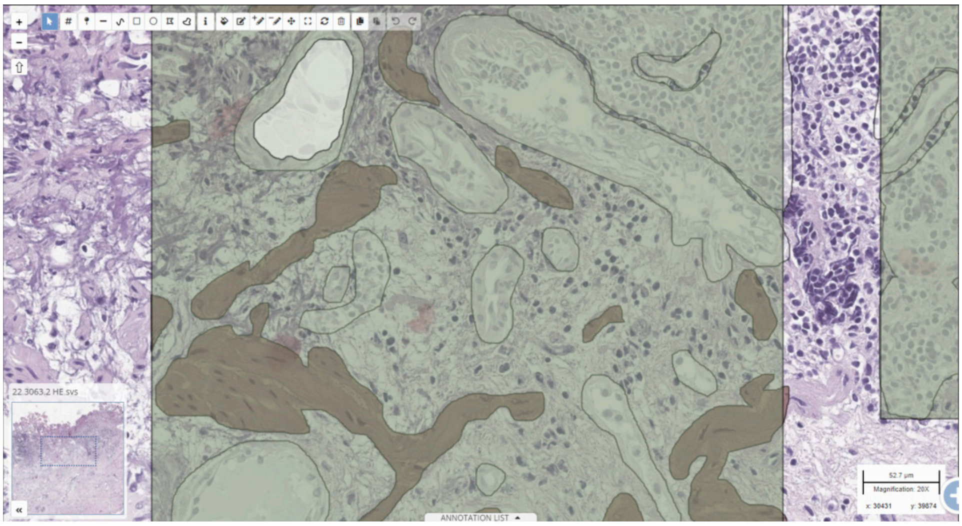

Figure S3. Vessels, stroma, smooth muscle, and one LVI (white). HE 20x

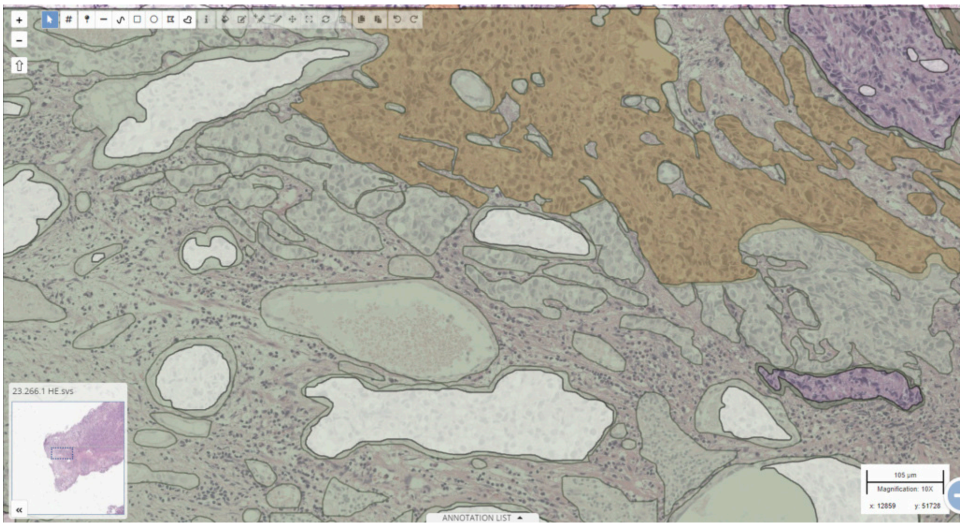

Figure S4. High grade tumor in orange; several LVI (white); stroma, vessels, and inflammation. HE 20x

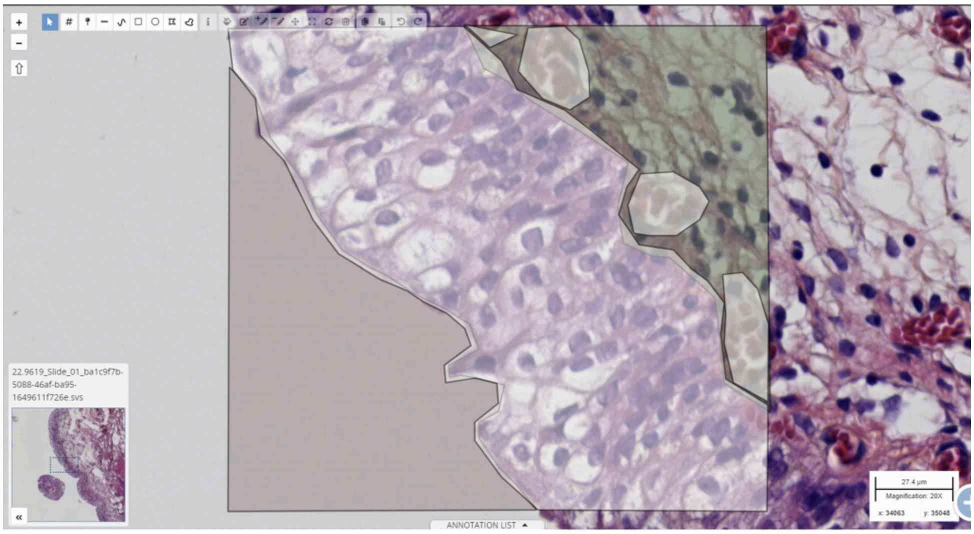

Figure S5. Reactive urothelium in white slightly overlapped on “no tissue” class (in gray); on the other side of urothelium — stroma and vessels. HE 40x

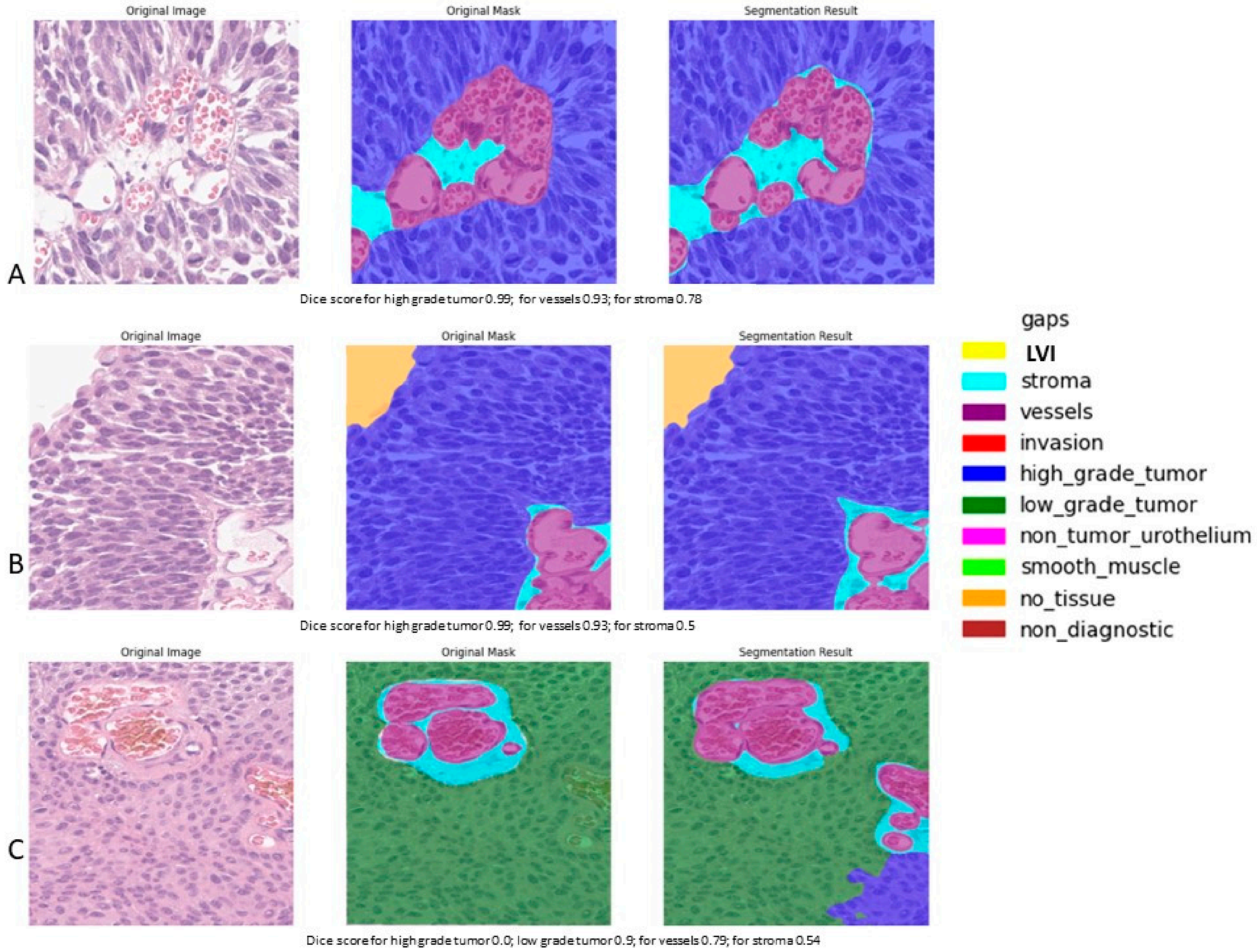

Figure S6. Several examples of automatic identification of a high-grade tumor. The original sample is depicted on the right, the central sample is the original mask (as it was annotated by the pathologist and further refined in order to eliminate the overlaps), and the right sample depicts the result offered by the algorithm. Both Dice score and IoU score are provided for each sample. Samples A-B were precisely segmented by our algorithm. The C Samples are theoretically less precisely labeled; when looking at the original image, it is is obvious that the stroma and vessels identified by the algorithm in the right margin of the segmentation image were in fact missed by the human annotator.

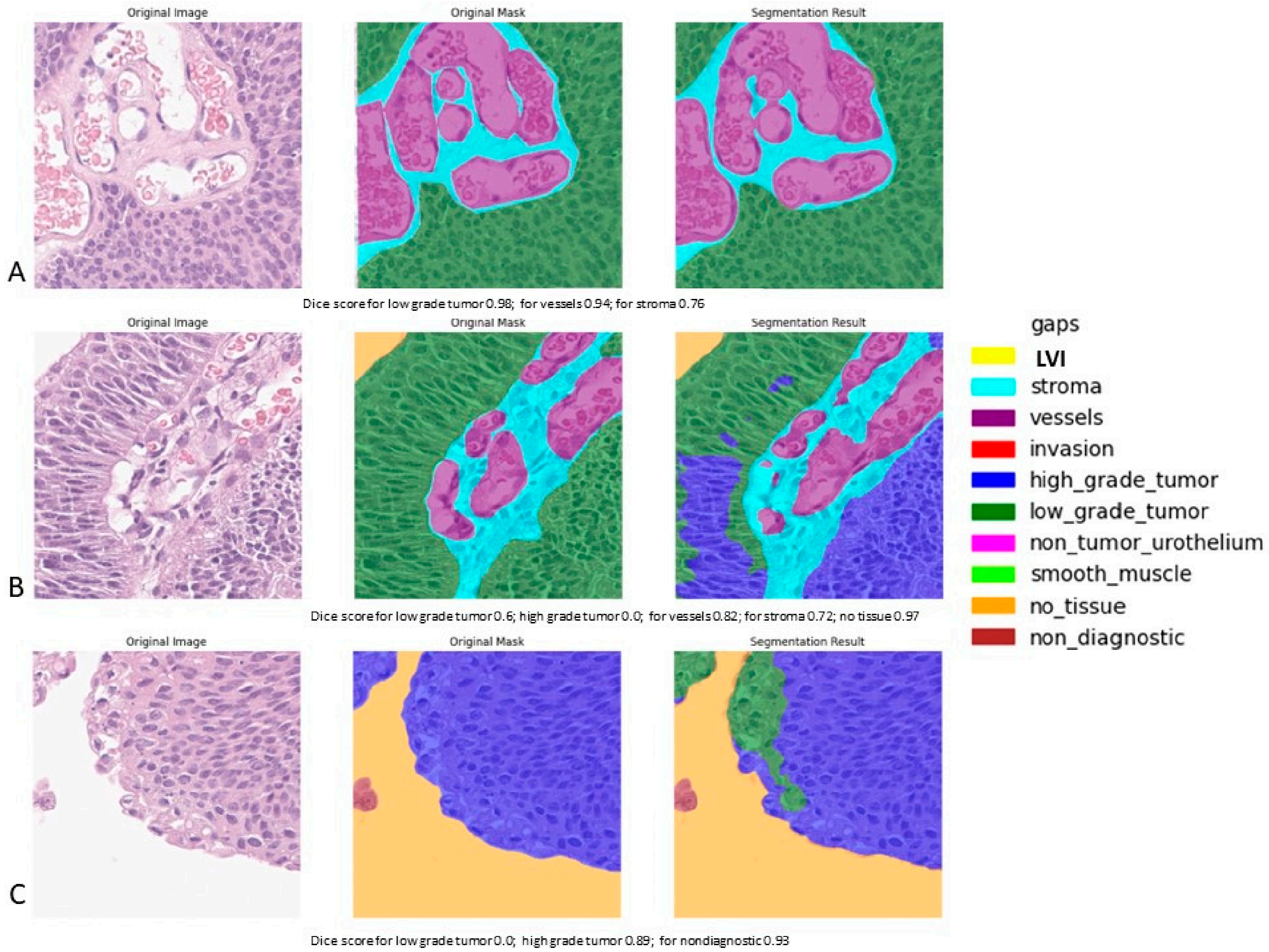

Figure S7. Several examples of automatic identification of a low-grade tumor. The original sample is depicted on the right, the central sample is the original mask (as it was annotated by the pathologist and further refined in order to eliminate the overlaps), and the right sample depicts the result offered by the algorithm. Both Dice score and IoU score are provided for each sample. Minute areas of high-grade or low-grade tumor (less than the dimension of one normal epithelial cell) have no importance for diagnosis and should be eliminated from the final heat-map based on a level of cut-off.

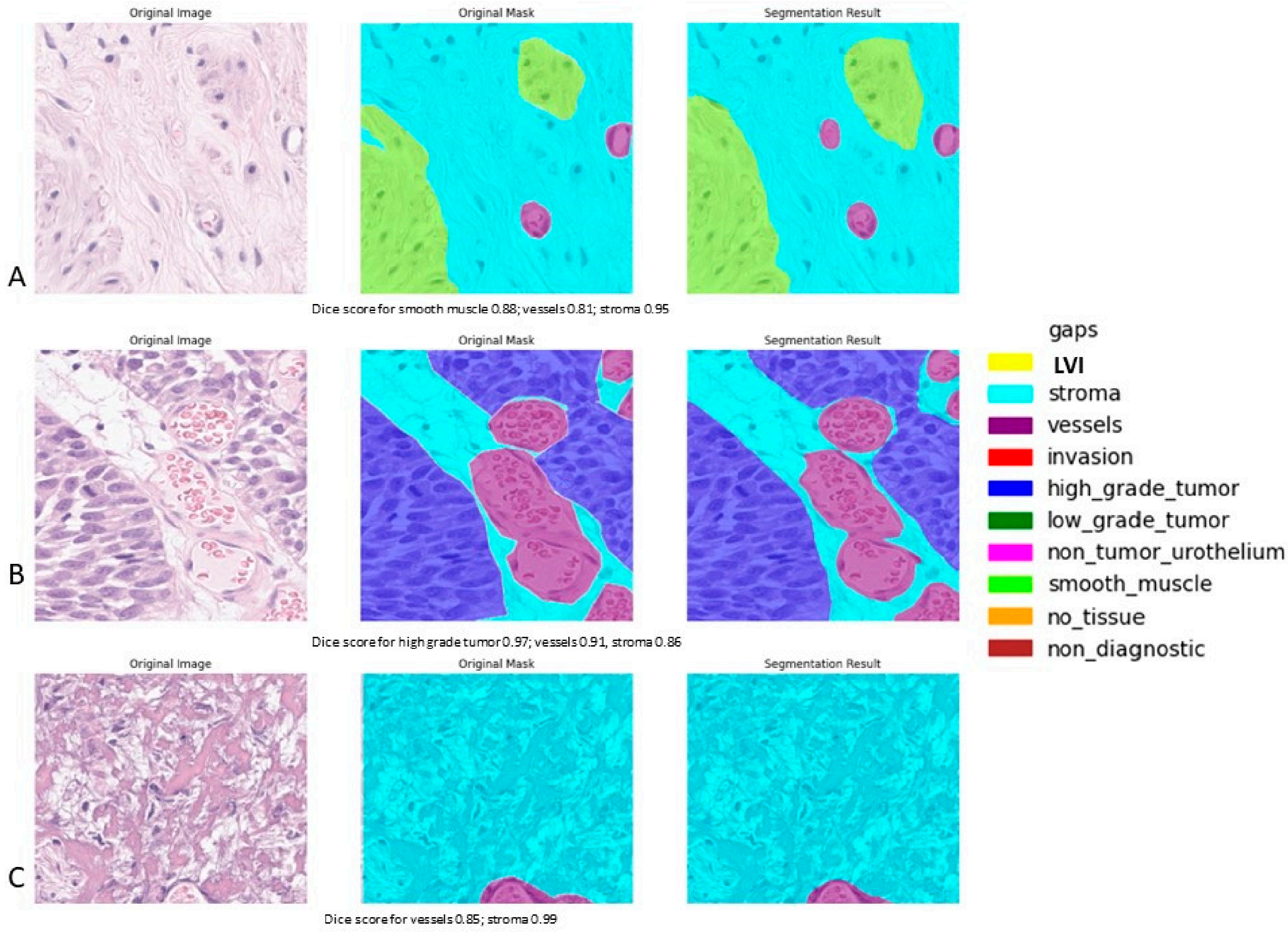

Figure S8. Several examples of automatic identification of smooth muscle, stroma, and vessels. The original sample is depicted on the right, the central sample is the original mask (as it was annotated by the pathologist and further refined in order to eliminate the overlaps), and the right sample depicts the result offered by the algorithm. Both Dice score and IoU score are provided for each sample. The algorithm performs superbly when evaluating stroma, vessels, and smooth muscle; the metrics were quite low due to the method of evaluation (pixel per pixel) and not the units identified.
